# The synthesis of *n*-caproate from lactate: a new efficient process for medium-chain carboxylates production

**DOI:** 10.1038/srep14360

**Published:** 2015-09-25

**Authors:** Xiaoyu Zhu, Yong Tao, Cheng Liang, Xiangzhen Li, Na Wei, Wenjie Zhang, Yan Zhou, Yanfei Yang, Tao Bo

**Affiliations:** 1 Key Laboratory of Environmental and Applied Microbiology, Chinese Academy of Sciences; Environmental Microbiology Key Laboratory of Sichuan Province, Chengdu Institute of Biology, Chinese Academy of Sciences, Sichuan, 610041, PR China; 2 College of Life Science, Sichuan University, Sichuan, 610041, PR China

## Abstract

A unique microbiome that metabolizes lactate rather than ethanol for *n*-caproate production was obtained from a fermentation pit used for the production of Chinese strong-flavour liquor (CSFL). The microbiome was able to produce *n*-caproate at concentrations as high as 23.41 g/L at a maximum rate of 2.97 g/L/d in batch trials without in-line extraction. Compared with previous work using ethanol as the electron donor, the *n*-caproate concentration increased by 82.89%. High-throughput sequencing analysis showed that the microbiome was dominated by a *Clostridium* cluster IV, which accounted for 79.07% of total reads. A new process for *n*-caproate production was proposed, lactate oxidation coupled to chain elongation, which revealed new insight into the well-studied lactate conversion and carbon chain elongation. In addition, these findings indicated a new synthesis mechanism of *n*-caproate in CSFL. We believe that this efficient process will provide a promising opportunity for the innovation of waste recovery as well as for *n*-caproate biosynthesis.

*n*-Caproate, a medium-chain carboxylate (MCC), is a bio-based fuel precursor and valuable industrial commodity. *n*-Caproate has emerged as a new end product of the carboxylate platform in which waste is converted to valuable chemicals by a microbiome^1^. With the growing demand for the development of sustainable waste treatment, *n*-caproate, which is characterized by high-energy density and hydrophobicity, has been chosen as a more favourable end product than other valuable products, *e.g.*, methane, ethanol and short chain carboxylates (SCCs)^2^,^3^,^4^,^5^,^6^,^7^,^8^,^9^. For example, a laboratory-scale energy system that substitutes *n*-caproate for bio-ethanol derived from cellulosic feedstock has been established to circumvent the fossil fuel consumption for ethanol distillation^4^,^5^. A new anaerobic digestion technology, which converts municipal solid waste to valuable *n*-caproate, has also been reported^3^,^6^,^7^.

The best-known bio-pathway for *n*-caproate production is chain elongation, a reversed β-oxidation pathway in which SCCs, *e.g.*, acetate and butyrate, are elongated with two-carbon units, acetyl-CoA, derived from a reduced substrate^8^,^9^,^10^,^11^,^12^. Ethanol is regarded as the most efficient reduced substrate for *n*-caproate synthesis by the bacterium *Clostridium kluyveri*, which has been identified in many studies^1^,^4^,^5^,^6^,^8^,^9^,^10^,^11^,^12^. Without *in situ* products removal, the *n*-caproate concentration can reach up to 12.8 g/L via this pathway^13^. Other substrates, *e.g.*, hydrogen and electrons, have been found to form *n*-caproate at concentrations of 0.98–1.75 and 0.74 g/L^14^,^15^,^16^. In addition, *Clostridium* sp. BS-1, *Kluyveromyces marxianus* and *Megasphaera elsdenii* have been found to utilize D-galactitol, galactose and sucrose, producing 0.98, 0.15 and 4.69 g/L of *n*-caproate, respectively^17^,^18^,^19^. The yields of *n*-caproate derived from these substrates are lower than those derived from ethanol so far, and therefore, *n*-caproate production investigations have focused on the ethanol conversion pathway^2^,^3^,^4^,^5^,^6^,^7^,^8^,^9^. In a typical example of the new anaerobic digestion process for *n*-caproate recovery, municipal solid waste is converted to *n*-caproate through the addition of ethanol to the upflow anaerobic filter^3^,^7^,^8^,^9^.

The fermentation pit used for Chinese strong-flavour liquor (CSFL) production is a unique artificial environment for *n*-caproate production that contains up to 20.0 g/kg (dry pit mud) *n*-caproate^20^. Because liquor fermentation is an ethanol-producing process, it is generally believed that the *n*-caproate in CSFL is synthesized from ethanol^21^. However, in our previous study, we observed that the significant increase in *n*-caproate production was accompanied by a continuous decline in lactate during the development of CSFL fermentation^20^. It was also found that the concentration of lactate is several times higher than that of ethanol in the fermentation pit^20^. Although lactate can provide acetyl-CoA for *n*-butyrate production via reversed β-oxidation^22^,^23^,^24^,^25^, it has never been considered as a feedstock for *n*-caproate production, as only trace amounts of *n*-caproate could be produced in previous studies^26^,^27^.

The present study found that lactate can serve as a sole carbon and energy source for *n*-caproate production by a unique microbiome obtained from the pits, whereas a small amount of *n*-caproate was produced when the microbiome was fed ethanol. The capacity of the microbiome to produce *n*-caproate was then tested in lactate-containing medium. Finally, possible processes for the remarkably high *n*-caproate production by the unique microbiome from lactate were discussed.

## Results

### *n*-Caproate Production and Substrate Identification

Mature pit mud (with a pit age of more than 20 years, detailed in the Supplementary Information) was inoculated into the 5 times diluted “yellow water”, diffusate from the fermentation mash, which consisted of mainly lactate, ethanol and glucose (detailed in the Supplementary Information). After more than 90 days of acclimation, the semi-continuously operated reactor (SCOR) achieved stable *n*-caproate production (see Supplementary Information, Fig. S2). According to literature, *n*-caproate is usually produced from ethanol. However, interestingly, the simultaneous accumulation of ethanol and *n*-caproate was observed during the acclimation period, whereas lactate, the other main component, was usually under the detection limit. In this paper, the mechanism for significant *n*-caproic acid production was then investigated through the substrate identification.

**Figure 1:**
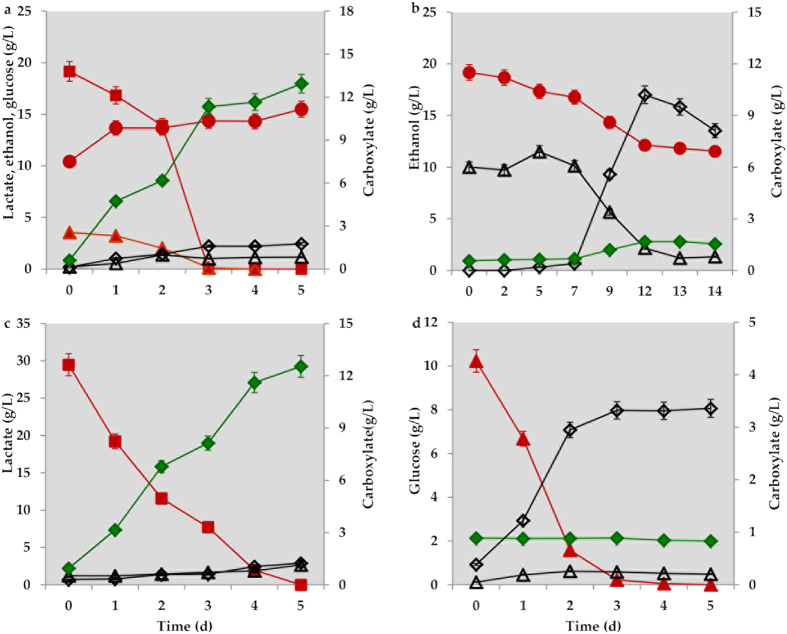
The production of *n*-caproate using different substrates (a: yellow water as control, b: ethanol, c: lactate, d: glucose) by the unique microbiome. ● ethanol (red); ▲ glucose (red); ■ lactate (red); Δ acetate (black); ♢ *n-*butyrate (black); ♦ *n-*caproate (green).

As shown in Fig. 1a, when yellow water was used as the carbon source, the *n*-caproate concentration increased from 0.61 to 12.93 g/L on day 5, resulting in an average volumetric *n*-caproate production rate of 2.46 g/L/d. The ethanol concentration increased from 10.42 to 15.50 g/L. The ethanol produced may be derived from other carbohydrates in the “yellow water”. By contrast, both lactate and glucose were depleted to concentrations below the detectable limits from initial concentrations of 19.15 and 3.54 g/L, respectively. Acetate and *n*-butyrate increased from 0.20 g/L to 1.16 and 2.44 g/L, respectively (Fig. 1a).

When ethanol was used as the sole reduced substrate, the obtained *n*-caproate level was only 1.12 g/L, whereas 10.20 g/L of *n*-butyrate was achieved on day 12 (Fig. 1b). Then, the *n*-butyrate concentration decreased once the acetate was exhausted. The consumption of ethanol in two weeks was only 7.34 g/L, leaving a large quantity of ethanol remaining.

The other two main components in “yellow water”, lactate and glucose, were then examined. When lactate served as the sole carbon and energy source, the *n*-caproate concentration reached up to 12.54 g/L in 5 days (Fig. 1c), and both acetate (0.62 g/L) and *n*-butyrate (0.95 g/L) were detected. Although some bacteria can convert glucose to *n*-caproate^28^, in our experiment, glucose was exhausted in 3 days predominantly by the production of *n*-butyrate (3.36 g/L) and acetate (0.49 g/L). This result indicates that butyrate-producing bacteria may take advantage of the limited glucose (Fig. 1d). The selectivity of *n*-caproate was 81.36% in the lactate reactor, which was 8.88 times higher than that in the ethanol reactor (see Supplementary Information, Table S1). These results show that the unique microbiome utilizes lactate predominantly for *n*-caproate production, rather than ethanol or glucose. As none of bacteria has been reported to produce large amount of *n*-caproate as major product from lactate, it would be interesting to investigate the community of the unique microbiome.

### Community Composition Analysis

**Figure 2:**
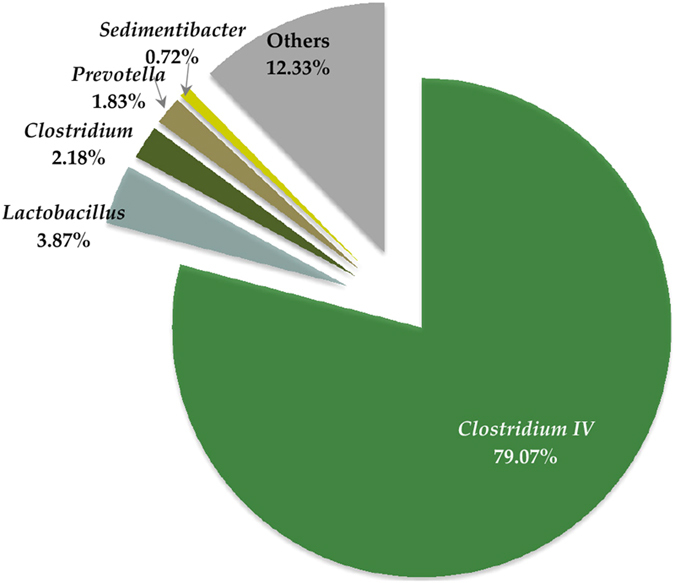
The relative abundance levels of genera in the prokaryotic community in the acclimated bioreactors (SCOR) at day 90.

As shown in Fig. S2, caproate production (in reactor SCOR) maintained relatively stable after 30 days, so the microbial communities of the SCOR on day 30 and 90 were investigated to analyse their variation during the long term acclimation. It was found that *Clostridium* cluster IV (the family of *Ruminococcaceae*) was always the dominant group. On day 30, *Clostridium* cluster IV made up 46.54% of the total 16S rRNA sequences (see Supplementary Information, Table S2), whereas 79.07% of the 16S rRNA gene sequences were affiliated with *Clostridium* cluster IV on day 90 (Fig. 2). The relative abundance of *Clostridium* cluster IV increased 1.68 times after 60 days. Consequently, the relative abundance of other genera, mainly *Lactobacillus* and *Clostridium*, decreased significantly to 3.87 and 2.18%, respectively (Table S2). *Lactobacillus* belongs to the *Lactobacillaceae* family, which displays a relatively simple carbon and energy metabolism in which lactose and other carbohydrates are converted to the reduced substrate lactate. *Prevotella* and *Sedimentibacter* made up 1.83 and 0.73% of the total genera, respectively. Methanogens, which may compete with *n*-caproate-producing bacteria for acetate, the carbon bone of *n*-caproate^4^,^6^, exhibited a very low relative abundance of 0.30% (see Supplementary Information, Table S2). Moreover, the two genera of methanogens were hydrogenotrophic methanogens. The average methane content was only 0.30% of the total gas (see Supplementary Information, Fig. S3), indicating that methanogens may be inhibited in the operating bioreactors. Low abundant populations (<0.50%) accounted for 12.33% of the total reads.

### Determination of the Capacity of *n*-Caproate Production from Lactate

**Figure 3:**
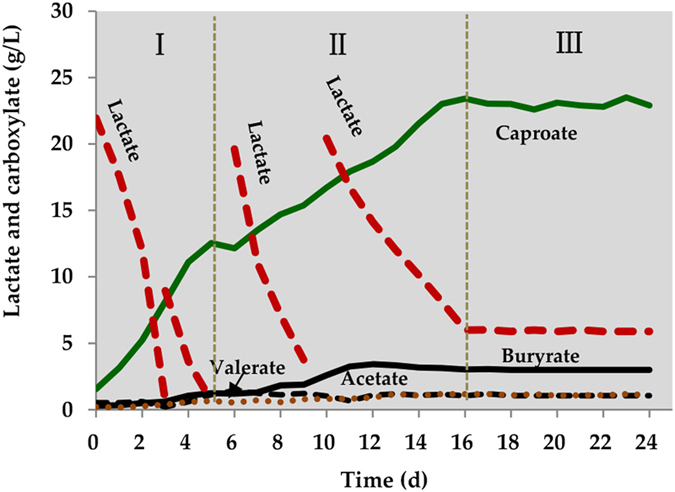
*n*-Caproate production and accumulation using lactate as the sole carbon source in batch experiments.

The capacity of *n*-caproate production of the unique microbiome enriched from the pit mud was investigated in a batch reactor (BR) fed lactate containing medium. Valuable *n*-caproate accumulated gradually with rapid lactate degradation (Fig. 3). The variation in *n*-caproate can be divided into three phases. In phase one, 26.62 g/L of lactate was rapidly converted to 10.99 g/L of *n*-caproate. The highest rate of *n*-caproate formation was 2.97 g/L/d. Without in-line product removal, the highest concentration of *n*-caproate obtained in previous studies is approximately 12.0 g/L^3^,^6^,^13^. However, in phase two, the concentration of *n*-caproate continuously increased to 23.41 g/L at an average rate of 1.08 g/L/d. In phase three, *n*-caproate production reached a plateau, with a concentration of approximately 23 g/L. Small quantities of acetate (0.53 g/L), *n*-butyrate (2.70 g/L) and *n-*valerate (0.88 g/L) were produced, resulting in high selectivity for *n*-caproate (84.74%), which was similar to the maximum selectivity of 85.0% using ethanol as the reduced substrate^9^. The hydrogen content gradually decreased to below the detection limit, which resulted in hydrogen pressure, an important factor that prevents *n*-caproate oxidation^24^, at a very low level. Indeed, *n*-caproate oxidation was not observed. Moreover, lactate and other products did not change significantly. Microbial community analysis of the BR showed that *Clostridium* cluster IV was still the dominant population, reaching a relative abundance of 52.39% in the microbiome (Fig. S4). The other two major populations were *Lactobacillus* and *Clostridium*. The cluster analysis (CA) at the order and genus levels indicated highly similarity of the microbial communities between BR and SCOR (Fig. S5). These findings confirmed the reproducibility of this unique microbiome.

## Discussion

In the present study, the unique microbiome was dominated by the *Clostridium* cluster IV (Fig. 2). Many lactate-utilizing, butyrate producing bacteria belong to *Clostridium* clusters IV and XIVa, particularly the latter^29^,^30^,^31^,^32^. Phylogenetic analysis based on 16S rRNA gene showed that most dominant OTUs of the unique microbiome were more closely related to the species of the *Clostridium* cluster IV (similarity of 92.2–95.7%) than other lactate-utilizing bacteria, *e.g.,* the species of *Clostridium* cluster XIVa (Fig. S6). So far, individual species classified in *Clostridium* cluster IV has been reported to be able to produce *n*-caproate from saccharides^17^, but none of the species classified in *Clostridium* cluster IV or XIVa has been found to synthesize *n*-caproate from lactate. However, our previous study on the changes in the communities in pit mud from different-aged pits demonstrated that *Clostridium* cluster IV has a positive correlation with *n*-caproate formation^20^. Furthermore, *Clostridium* cluster IV only constituted 12.71% of the total microbiome in the inoculum (pit mud)^20^, but the percentage increased significantly to 79.07% within 90 days of acclimation (Fig. 2). Therefore, the high relative abundance of *Clostridium* IV in the unique microbiome and metabolic features of *Clostridium* cluster IV members suggest that *Clostridium* cluster IV may play an important role in the use of lactate and in the production of *n*-caproate. *Lactobacillus* was the second most abundant group in the unique microbiome. The function of *Lactobacillus* is to ferment carbohydrates, *e.g.,* glucose to lactate. Thus, *Lactobacillus* may make little contribution to the lactate to *n*-caproate conversion. *Clostridium* accounted for 2.18% of the total 16S rRNA sequences. Because the best known species *C. kluyveri*, which use ethanol for caproate production belongs to this taxa, *Clostridium* may involve in the ethanol to *n*-butyrate and *n*-caproate conversion. *Prevotella* accounted for 1.83% of the total bacteria. This genus can produce *n*-butyric acid from lactic acid^33^.

During the lactate conversion process, small amounts of acetate and *n*-butyrate were observed (Fig. 3). This observation suggests that these metabolites may be intermediate products in *n*-caproate synthesis or end products of other less-abundant bacteria, *e.g.*, *Prevotella*. Acetate production can be explained via lactate oxidation^22^,^23^,^31^,^32^. The *n*-butyrate may be derived from lactate oxidation coupled to chain elongation (or reverse β-oxidation), in which acetate elongates its carbon chain with acetyl-CoA^22^,^23^,^24^,^25^,^32^. Ethanol oxidation coupled to chain elongation is one of most efficient processes for *n*-caproate generation. However, in the present study, when the sole electron donor fed was ethanol, low concentrations of *n*-caproate were detected (<2.0 g/L), but *n*-butyrate was the most common metabolite (Fig. 1b). Therefore, the ethanol oxidation coupling chain elongation was likely not the main pathway for *n*-caproate production in our experiment. Hydrogen is another electron donor used to form caproate because acetate can be reduced by hydrogen to ethanol, and subsequently, *n*-butyrate and *n*-caproate are produced from acetate with ethanol as the electron donor^25^,^34^,^35^. Because ethanol was not the main contributor to *n*-caproate production in our study, it seemed unfeasible that the unique microbiome using hydrogen as electron donor for caproate formation. Therefore, it was hypothesized that a new process may be responsible for the formation of *n*-caproate.

**Figure 4:**
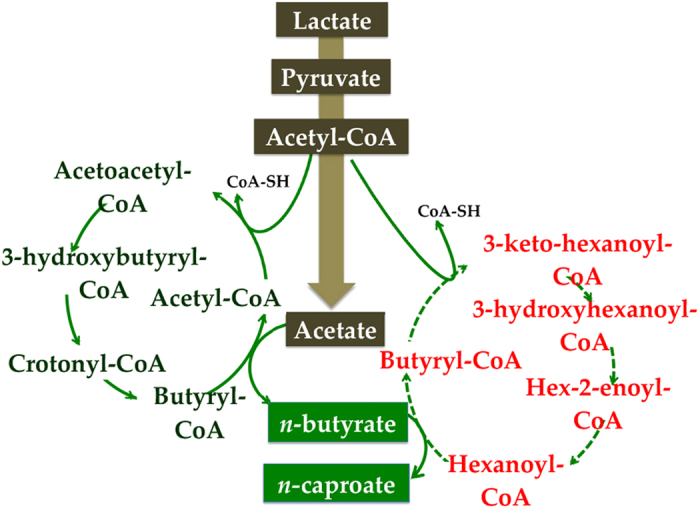
Proposed process for *n-*caproate formation from lactate. Because the corresponding genes of the enzymes that catalyse *n*-caproate formation were unknown, the *n*-caproate formation portion was presumed according to refs 24,36 and marked with a dashed line.

Lactate is another important energy-rich, reduced compound that can provide the necessary acetyl-CoA for butyrate production via chain elongation^32^. Seedorf *et al.* found that the enzymes that catalyse butyrate production have a function in hexanoyl-CoA for caproate formation^12^. Taken together, these data suggest that *n*-caproate production from lactate may be analogous to the process of ethanol oxidation/reverse β-oxidation, and a new process for *n*-caproate production was proposed: lactate oxidation coupled to reverse β-oxidation (Fig. 4).

In this proposed process, lactate is oxidized to acetate via pyruvate. Additionally, lactate oxidation provides the necessary acetyl-CoA for the acetate to elongate its carbon length to form *n-*butyrate. Subsequently, another acetyl-CoA molecule derived from lactate enters into the circle of reverse β-oxidation to form the key intermediate hexanoyl-CoA. Finally, the condensation of hexanoyl-CoA and butyrate leads to the production of *n*-caproate. During the whole process, acetyl-CoA, energy and reducing equivalents were all derived from lactate. Based on the metabolic process hypothesized, the n-caproate formation can be derived by three coupled reactions shown in Eqs (1)–(3):


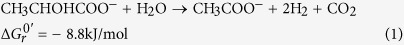



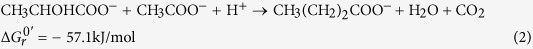



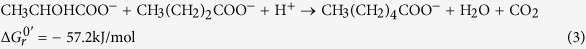


Eq. (1) describes the anaerobic oxidation of lactate to acetate, H_2_ and CO_2_, which leads to the synthesis of ATP. Eqs (2) and (3) describe the dehydrogenation of lactate, leading to the formation of *n*-butyrate from lactate and acetate and the formation of *n*-caproate from lactate and *n*-butyrate. The combination of Eqs (1) to (3), 2, 3 gives Eq. (4), which describes *n*-caproate formation via lactate oxidation coupled with reverse β-oxidation.


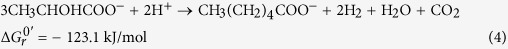


The overall conversion is thermodynamically favourable under standard conditions with a Gibbs free energy change of −123.1 kJ/mol. The Eq. 4 indicated that every 3 mol of lactate generates 1 mol of *n*-caproate. As shown in Fig. 1c, 328 mmol of lactate produced 99 mmol of *n*-caproate, which satisfies the speculated Eq. 4 well.

According to the thermodynamic analysis and our results, it appears that there is no biochemical barrier in the conversion of lactate to *n*-caproate, but the known lactate-utilizing bacteria, such as *Megasphaera elsdenii* and *Clostridium acetobutylicum*, chiefly produce *n*-butyric acid, as shown in previous studies^22^,^23^,^24^,^25^,^26^,^31^,^37^. These bacteria are isolated predominantly from the human intestinal tract and rumen where the pH is usually neutral and lactate can be metabolized rapidly. In contrast, the unique microbiome was evolved from an acidic environment (pH 3.0–6.0) containing up to 166 g/kg of lactate^20^. The acidic environment and high lactate accumulation may be beneficial for the evolution of lactate-utilizing and *n*-caproate-producing bacteria.

Many researchers found that the production of *n*-caproate is hardly to exceed 12 g/L without *in-situ* product removal, even though the pH is higher than the pKa of the *n*-caproic acid^3^,^4^,^5^,^6^,^7^,^8^,^9^,^13^. For examples, Steinbusch *et al.* used ethanol as electron donor to produce *n*-caproate at pH 7.0. The highest concentration they obtained was 8.27 g/L^6^. Roddick and Britz observed that *n*-caproate production from glucose can only be accumulated to 11.4 g/L in batch reactor (pH 7.0)^38^. Choi *et al.* used sucrose to produce 8.19 g/L of *n*-caproate (pH 7.0)^18^. It has been considered that the solubility and toxicity of *n*-caproate can restrict its bio-production^3^,^4^,^5^,^6^,^7^,^8^,^9^,^13^. By contrast, in the similar batch mode experiment without in-line product removal, the yield of *n*-caproate could reach up to 23.41 g/L in our study, which was nearly two times higher than reported results. Hence, this new process of lactate to *n*-caproate conversion is clearly a breakthrough for *n*-caproate synthesis.

As the lactate pathway enables the rapid disposal of reducing equivalents, lactate fermentation usually dominates primary fermentation in mixed cultures when high concentrations of easily degradable substrates, such as food waste, are available^11^,^39^,^40^. Recent trends further demonstrated that lignocellulosic substrates, *e.g.,* corn stalk and sugarcane waste, can also be used for large amount of lactate production^41^,^42^. In light of these researches, we provide a new strategy that recover *n*-caproate from lactate containing fermentation products of waste or agricultural residues which are largely available. It is important to investigate how to combine lactate fermentation with *n*-caproate production in the future. Given that *n*-caproate recovery from waste requires sufficient ethanol addition in the current process, the new process of *n*-caproate production from lactate might become an attractive candidate in biotechnology to recover low grade organic matters to high value-added *n*-caproate without exogenous energy substrate supply.

## Methods

### *n*-Caproate Formation from “Yellow Water”

“Yellow water” (detailed in the Supplementary Information) was diluted by a factor of five with distilled water. The diluted yellow water was boiled for 20 min and flushed with nitrogen gas for 20 min to remove oxygen. In total, 20 g of pit-mud inoculum (detailed in the Supplementary Information) was inoculated into 1.4 L of diluted yellow water in a 1.5-L SCOR (Fig. S1a). 50 mL of effluent was pumped out every day, and an equal volume of yellow water was fed, followed by a reaction period with pH controlled between 5.5 and 6.5 with 5 M HCl and 2 M NaOH. The temperature was maintained at (30 ± 1) °C. The experiment was performed in two parallel replicates.

### Identification of the Substrate for *n*-Caproate Generation

The inoculum was obtained from the effluent of a 1.5-L fermenter (SCOR) that had been fed with “yellow water” for 90 days. A volume of 50 mL of the inoculum described above was inoculated into glass bioreactors with a working volume of 1.0 L (Fig. S1b). The synthetic medium contained the following (g/L): 0.25 NH_4_Cl, 0.20 MgSO_4_·7H_2_O, 0.23 KH_2_PO_4_, 0.31 K_2_HPO_4_, 0.80 NaCl, 0.25 L-Cysteine-HCl·H_2_O, 1 mL vitamin solution (see Supplementary Information), 1 mL trace element solution (see Supplementary Information), and 1000 mL distilled water. In addition, 30.0 g/L lactate, 20.0 g/L ethanol (containing acetate 8 g/L), and 10.0 g/L glucose were fed into three groups of reactors, respectively. A control using yellow water as the carbon source was also set up, and a volume of 200 mL of yellow water was added into the basal medium described above. Each group was set up in triplicate. The medium was boiled for 20 min and flushed with nitrogen gas for 10 min. The pH was manually controlled between 6.0–6.5 with 5 M HCl and 2 M NaOH. The temperature was maintained at (30 ± 1) °C.

### Assessment of Capacity of the *n*-Caproate Production from Lactate

The experiment was performed in a batch reactor (BR) with a working volume of 1 L (Fig. S1b). Carbon felts (1.0 × 1.0 cm) were used to immobilize the microbiome. Lactate was used as the sole carbon source in the synthetic medium, which contained (per L) 5 mL of solution A, 2 mL of solution B, a certain amount of the carbon source (lactate), 1 mL of vitamin solution and 1 mL of trace element solution (see in Supplementary Information). Solution A contained the following per L: 10 g of MgSO_4_·7H_2_O, 4.5 g of CaCl_2_·H_2_O, 93.6 g of NH_4_Cl. Solution B contained the following per L: 29 g of KH_2_PO_4_ and 33 g of K_2_HPO_4_. The initial lactate concentration was approximately 20 g/L. The batch experiment ran for 4 cycles. In the first cycle, 950 mL fresh medium was injected into the reactor, and then a 50 mL culture withdrawn from the SCOR was inoculated, followed by a reaction period of 25 days with pH controlled. During the experimental period, lactate was fed into the reactor when it was exhausted. At the end of the cycle, all culture was pumped out, and then 1 L of fresh medium was injected and the next cycle began. The pH was manually controlled between 6.0–6.5 with 5 M HCl and 2 M NaOH. The temperature was maintained at (30 ± 1) °C.

### DNA Extraction and MiSeq Sequencing of 16S rRNA Gene Amplicons

Samples (50 mL) were withdrawn from the SCOR at days 30 and 90. An equal amount of sample was withdrawn from the BR at the end of the fourth cycle. All of these samples were centrifuged at 10,000 rpm for 10 min, and then pellets were used for genomic DNA extractions. The PowerSoil DNA Isolation Kit (MoBio Laboratories, USA) was used. DNA density and quality were checked using a NanoDrop Spectrophotometer. Extracted DNA was diluted to a concentration of 10 ng/μL and stored at −40 °C for downstream use. The universal primers 515F (5′-GTGCCAGCMGCCGCGGTAA-3′) and 806R (5′-GGACTACHVGGGTWTCTAAT-3′) with 10 nt barcodes were used to amplify the V4 hypervariable regions of 16S rRNA genes for next-generation sequencing using a Miseq sequencer^43^,^44^. PCR and MiSeq sequencing information are detailed in refs 43,44. The sequence data were processed using QIIME Pipeline–Version 1.8.0 (http://qiime.org/tutorials/tutorial.html). All sequence reads were trimmed and assigned to each sample based on their barcodes. Multiple steps were required to trim the sequences, such as the removal of sequences <150 bp and those with an average base quality score Q < 30. The phylogenetic affiliation of each 16S rRNA gene sequence was analysed by a RDP Classifier at a confidence level of 80% (http://pyro.cme.msu.edu/).

### Chemical Analysis

Liquid samples were withdrawn every day, centrifuged for 5 min at 10,000 rpm, diluted 20 times with distilled water and then sterilized using a 0.22-μm filter. Carboxylates (C1–C6), lactate, ethanol and glucose concentrations were determined by an Agilent 1260 Infinity liquid chromatography system (Agilent Technologies, USA) equipped with a high performance liquid chromatography (HPLC) column Hi-Plex H (300 × 6.5 mm) and a differential refraction detector (RID). H_2_SO_4_ (0.005 M) at a flow rate of 0.6 mL/min was used as the mobile phase. Gas production was estimated by measuring the water displacement. Hydrogen, carbon dioxide and methane analyses were performed using an Agilent 6890 gas chromatography (GC) system (Agilent Technologies, USA) with a thermal conductivity detector (TCD) and a 2-m stainless steel column packed with Porapak Q (50/80 mesh). The operating temperatures at the injection port, column oven, and detector were 100, 70, and 150 °C, respectively. Argon, at a flow rate of 30 mL/min, was used as the carrier gas.

### Calculations

Selectivity is the application of bioenergetics to estimation of yield efficient, showing the energy flow in metabolism of bacteria. The selectivity is defined as the concentration of electrons in the product formed divided by the net electrons consumed from the carbon and energy sources. Glucose and lactate contain 24 and 12 mol electrons per mol, respectively^45^. The detailed calculation is shown in ref. 3.

## Additional Information

**How to cite this article**: Zhu, X. *et al.* The synthesis of *n*-caproate from lactate: a new efficient process for medium-chain carboxylates production. *Sci. Rep.* **5**, 14360; doi: 10.1038/srep14360 (2015).

## Supplementary Material

Supplementary Information
