# Supplementary material for: The synthesis of n-caproate from lactate: a new efficient process for medium-chain carboxylates production
Source: Sci Rep. 2015 Sep 25;5:14360. doi: 10.1038/srep14360 (PMC4585908; doi:10.1038/srep14360)
Supplement: Supplementary Information [file srep14360-s1.pdf]

The synthesis of *n*-caproate from lactate, a new efficient process for medium chain carboxylate production

Xiaoyu Zhu[a], Yong Tao[a]*, Chen Liang[a], Xiangzhen Li[a], Na Wei[a], Wenjie Zhang[b], Yan Zhou[b], Yanfei Yang[a], Tao Bo[a].

[a.] Key Laboratory of Environmental and Applied Microbiology, Chinese Academy of Sciences; Environmental Microbiology Key Laboratory of Sichuan Province, Chengdu Institute of Biology, Chinese Academy of Sciences, Sichuan, 610041, PR China

[b.] College of Life Science, Sichuan University, Sichuan, 610041, PR China

* Corresponding author.

Yong Tao,

Tel.: +86-028-82890211

E-mail: taoyong@cib.ac.cn

Materials:

Chinese strong-flavor liquor brewing process

Chinese strong-flavored liquor (CSFL) is produced by traditional solid-state fermentation method, which has several thousand years of history in Chinese. In brief, fermentation materials (wheat, sorghum and corn) are put into a pit, in which the entire inner wall is covered with pit mud, which contains various flavor producing microbiomes. For every 2-3 months of airtight fermentation, fermentation diffusate (called "yellow water") is pumped out, and then fermentation mash are taken out for wine distilling. CSFL is famous for its unique flavor. The main flavor component is ethyl caproate, which is produced from *n*-caproic acid. The *n*-caproic acid concentration controls the quality of CSFL.

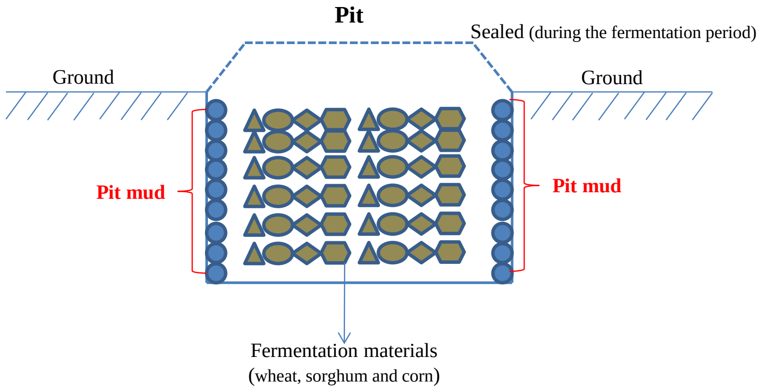

Inoculum

The pit mud acts as a source of inoculum and a habitat of microbes in the brewing fermentation pit for the CSFL production, in which microbes can produce various flavor components such as butyric acid, caproic acid and ethyl caproate. In this study, the initial inoculum was the pit mud of an old fermentation pit, which had been used to produce CSFL continuously for over 20 years. The pit is located in Mianzhu city,

Sichuan province, China.

"Yellow water"

A diffusate from fermentation mash, is brown and sticky muddy liquid produced from solid-fermentation of mixed materials including wheat, sorghum and corn. Dominated compositions of yellow water in this study were (g/L), lactic acid, 96.5 ± 4.2, ethanol, 50.5 ± 2.9, glucose, 15.6 ± 2.2.

Vitamin and trace elements solution

Vitamin for medium: vitamin B12, 100.00 mg; p-aminobenzoic acid 80.00 mg; D(+)-biotin 20.00 mg; nicotinic acid 200.00 mg; calcium pantothenate 100.00 mg; pyridoxine hydrochloride 300.00 mg; thiamine-HCl × 2 H_2O 200.00 mg; distilled water 1000.00 ml.

Vitamin for artificial wastewater: biotin 2.0 mg; folic acid 2.0 mg; pyridoxine hydrochloride 10.0 mg; thiamine·HCl 5.0 mg; riboflavin 5.0 mg; nicotinic acid 5.0 mg; calcium D-(+)-pantothenate 5.0 mg; vitamin B12 0.1 mg; p-aminobenzoic acid 5.0 mg; thioctic acid 5.0 mg; distilled water 1000.00 ml.

Trace elements solution: nitrilotriacetic acid 2.0 g; $MnSO_4 \cdot H_2O$ 1.0 g; $Fe(SO_4)_2(NH_4)_2 \cdot 6H_2O$ 0.8 g; $CoCl_2 . 6H_2O$ 0.2 g; $ZnSO_4 \cdot 7H_2O$ 0.2 mg; $CuCl_2 \cdot 2H_2O$ 20.0 mg; $NiCl_2 \cdot 6H_2O$ 20.0 mg; $Na_2MoO_4 \cdot 2H_2O$ 20.0 mg; Na_2SeO_4 20.0 mg; Na_2WO_4 20.0 mg; distilled water 1000.00 ml.

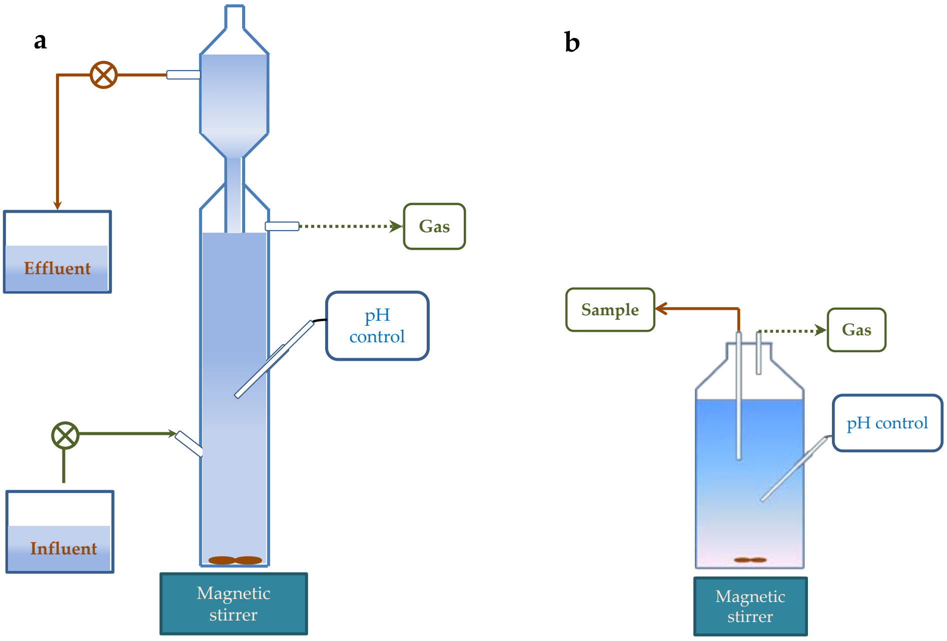

Figure S1 Schematic of the bioreactor setup: a, bioreactor fed with yellow water and operated semi-continuously(SCOR); b, bioreactor for substrates identification (batch reactor, BR).

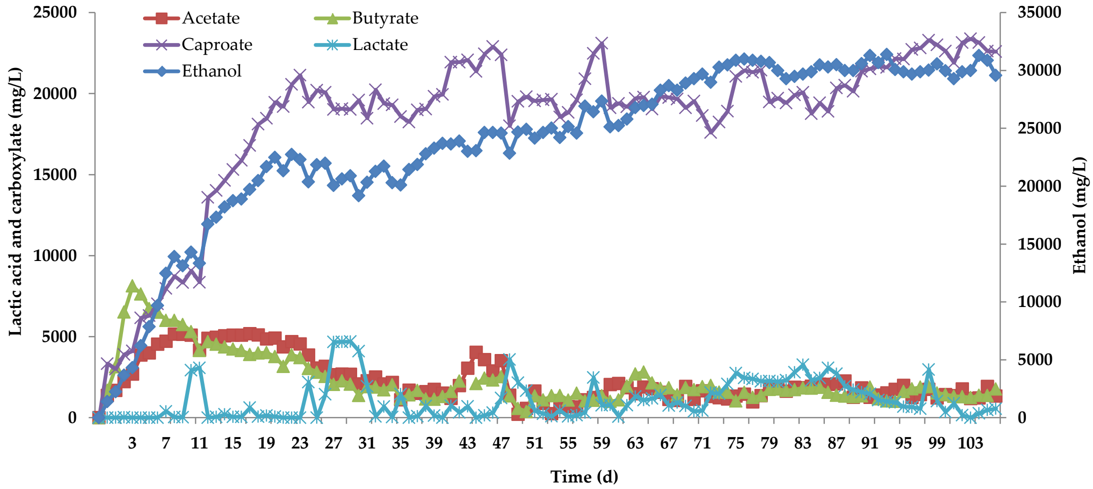

Figure S2 Dynamic changes of carboxylate, lactate, ethanol concentration during the acclimation period feeding with yellow water in reactor-SCOR.

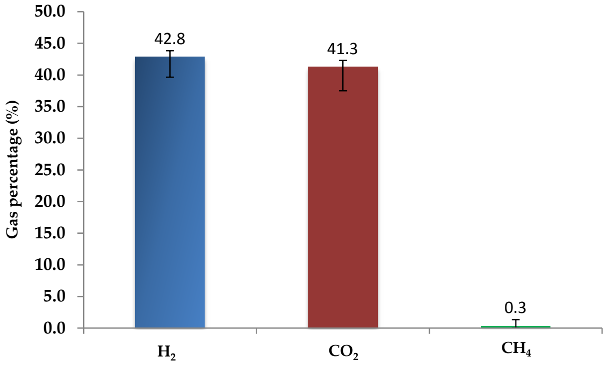

Figure S3 Average data of gas composition in the headspace of reactor (SCOR) during the acclimation period feeding with yellow water.

Table S1 Comparison of product selectivities (based on electrons) by using different reducing substrates

	products					
	formate [a]	acetate [a]	propionate [a]	butyrate [a]	valerate [a]	caproate [a]
ethanol [d]	n.d.[c]	- [b]	3.21	78.59	4.62	9.16
lactic acid	n.d.[c]	2.10	n.d.[c]	5.47	3.10	81.36
glucose	4.81	2.15	n.d. [c]	29.75	n.d. [c]	n.d. [c]

[a] : the unit was %.

[b]: acetic acid was exhausted at the final day.

[c]: concentration was always lower than the detecting line.

[d]: fed with acetate at the same time

Table S2 The relative abundance of prokaryotic community in the fermentation reactor SCOR #1 and #2 on day 30 and 90 at genus levels.

Family and genus	30 days			90 days		
	#1	#2	average	#1	#2	average
Ruminococcaceae	53.48	40.24	46.86 ± 9.36	77.91	80.55	79.23 ± 1.87
Clostridium IV	53.20	39.88	46.54 ± 9.42	77.74	80.41	79.07 ± 1.89
Lactobacillaceae	9.93	17.21	13.57 ± 5.15	4.24	3.55	3.90 ± 0.49
Lactobacillus	9.93	17.21	13.57 ± 5.15	4.21	3.52	3.87 ± 0.49
Clostridiaceae	5.91	18.76	12.34 ± 9.09	3.52	3.38	3.45 ± 0.10
Clostridium	3.19	16.93	10.06 ± 9.72	2.26	2.10	2.18 ± 0.11
Caloramator	2.07	0.09	1.08 ± 1.40	0.18	0.14	0.16 ± 0.03
Lachnospiraceae	2.16	2.30	2.23 ± 0.10	0.18	0.22	0.20 ± 0.03
Coprococcus	0.97	1.26	1.12 ± 0.21	0.14	0.13	0.14 ± 0.01
Prevotellaceae	0.04	0.02	0.03 ± 0.01	1.68	1.98	1.83 ± 0.21
Prevotella	0.04	0.02	0.03 ± 0.01	1.68	1.98	1.83 ± 0.21
Tissierellaceae	2.14	0.12	1.13 ± 1.43	1.36	1.28	1.32 ± 0.06
Tissierella	1.87	0.07	0.97 ± 1.27	0.14	0.11	0.13 ± 0.02
Sedimentibacter	0.05	0.02	0.04 ± 0.02	0.77	0.68	0.72 ± 0.06
Methanobacteriaceae	0.35	0.02	0.19 ± 0.23	0.36	0.29	0.33 ± 0.05
Methanobrevibacter	0.02	0.01	0.02 ± 0.01	0.21	0.15	0.18 ± 0.04
Methanobacterium	0.33	0.02	0.18 ± 0.22	0.14	0.14	0.14 ± 0.00

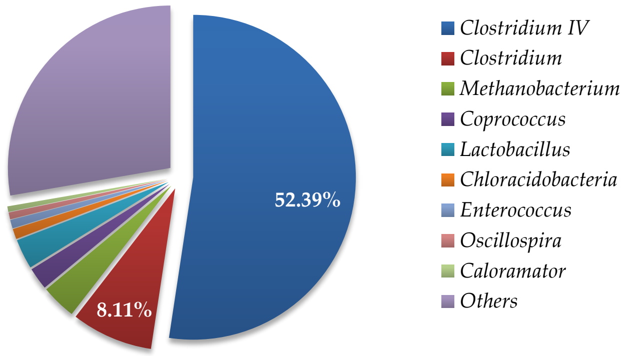

Figure S4. The relative abundance of prokaryotic community in batch reactors (BR) at genus levels

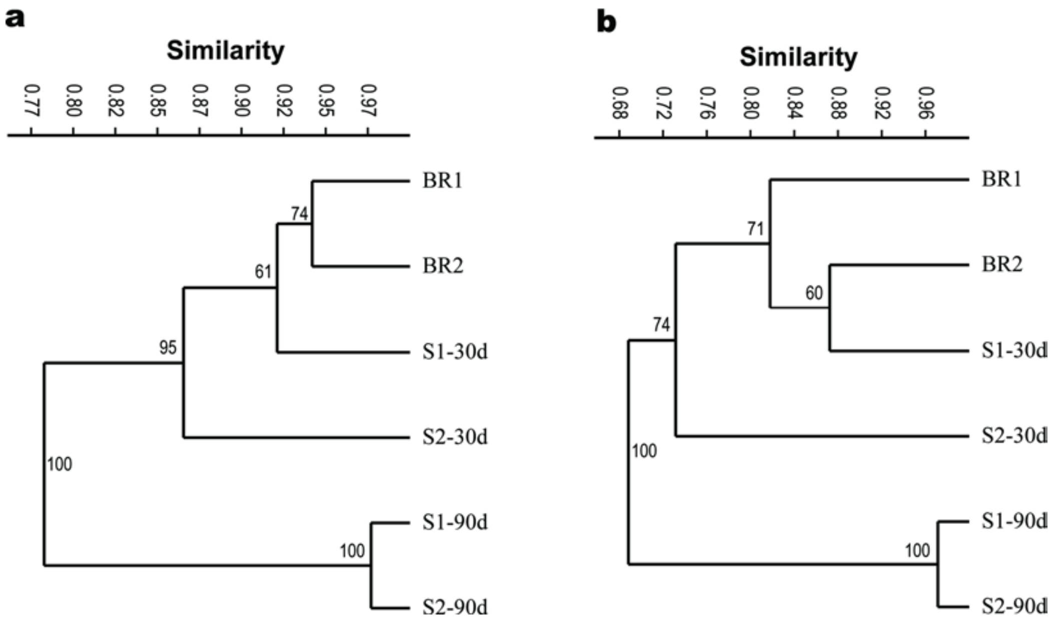

Figure S5. Cluster analysis (CA) of microbial communities in semi-continuously operated reactor (SCOR) and batch reactor (BR) conducted using the Bray–Curtis distance in PAST based on at order level (a) and genus level (b). The numbers at the nodes indicate the level of bootstrap of 1000 replications.

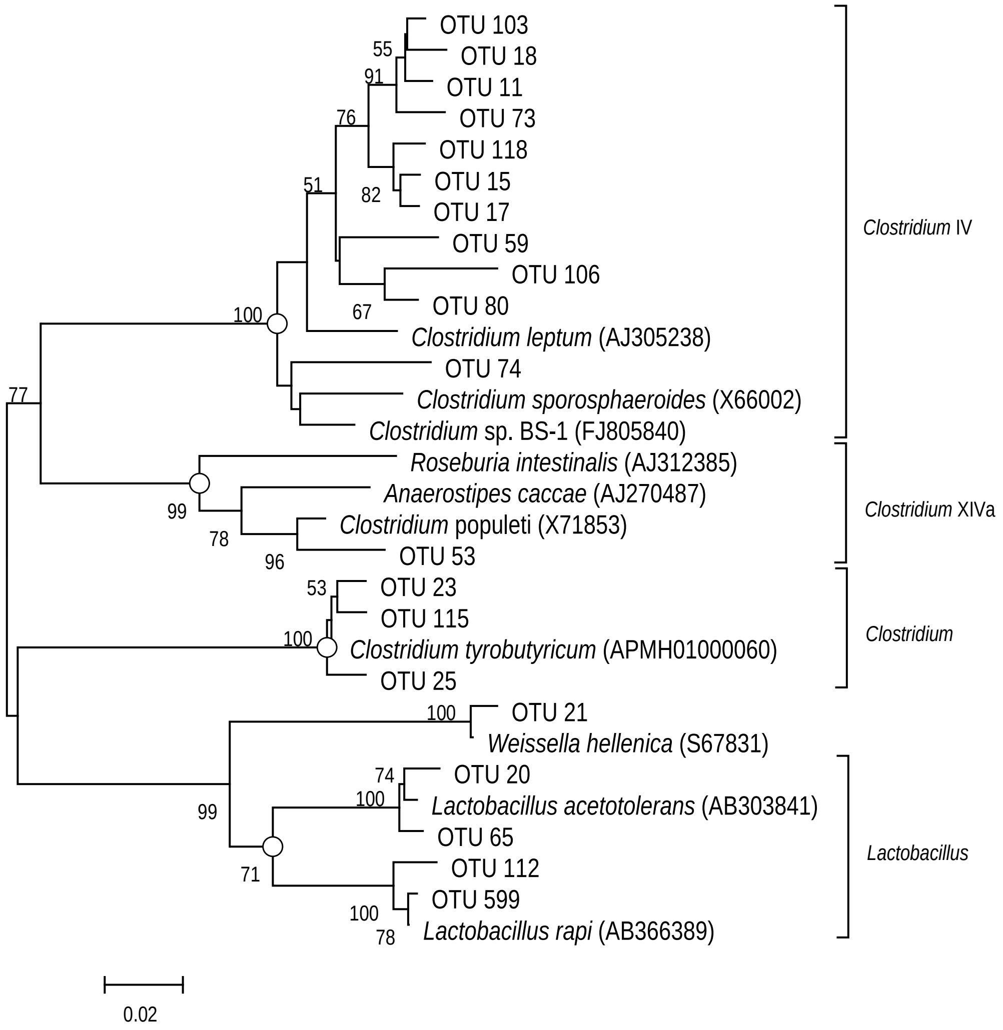

Figure S6. Phylogenetic tree of the abundant OTUs of the microbiome and related species based on 16S rRNA gene sequence similarity. GenBank accession numbers of the 16S rRNA gene sequences are given in brackets. Bootstrap values (expressed as percentages of1,000 replications) greater than 50% are shown at nodes. Bar,0.02 substitutions per nucleotide position.
